# Large scale changes in the transcriptome of *Eisenia fetida* during regeneration

**DOI:** 10.1371/journal.pone.0204234

**Published:** 2018-09-27

**Authors:** Aksheev Bhambri, Neeraj Dhaunta, Surendra Singh Patel, Mitali Hardikar, Abhishek Bhatt, Nagesh Srikakulam, Shruti Shridhar, Shamsudheen Vellarikkal, Rajesh Pandey, Rijith Jayarajan, Ankit Verma, Vikram Kumar, Pradeep Gautam, Yukti Khanna, Jameel Ahmed Khan, Bastian Fromm, Kevin J. Peterson, Vinod Scaria, Sridhar Sivasubbu, Beena Pillai

**Affiliations:** 1 CSIR – Institute of Genomics and Integrative Biology, Mathura Road, New Delhi, India; 2 Academy of Scientific & Innovative Research (AcSIR), Mathura Road, Delhi, India; 3 CSIR Ayurgenomics Unit - TRISUTRA, CSIR-IGIB, New Delhi, India; 4 Lifecode Technologies, New Delhi, India; 5 Science for Life Laboratory, Department of Molecular Biosciences, The Wenner-Gren Institute, Stockholm University, Stockholm, Sweden; 6 Department of Biological Sciences, Dartmouth College, Hanover, New Hampshire, United States of America; Dokuz Eylul Universitesi, TURKEY

## Abstract

Earthworms show a wide spectrum of regenerative potential with certain species like *Eisenia fetida* capable of regenerating more than two-thirds of their body while other closely related species, such as *Paranais litoralis* seem to have lost this ability. Earthworms belong to the phylum Annelida, in which the genomes of the marine oligochaete *Capitella telata* and the freshwater leech *Helobdella robusta* have been sequenced and studied. Herein, we report the transcriptomic changes in *Eisenia fetida* (Indian isolate) during regeneration. Following injury, *E*. *fetida* regenerates the posterior segments in a time spanning several weeks. We analyzed gene expression changes both in the newly regenerating cells and in the adjacent tissue, at early (15days post amputation), intermediate (20days post amputation) and late (30 days post amputation) by RNAseq based de novo assembly and comparison of transcriptomes. We also generated a draft genome sequence of this terrestrial red worm using short reads and mate-pair reads. An in-depth analysis of the miRNome of the worm showed that many miRNA gene families have undergone extensive duplications. Sox4, a master regulator of TGF-beta mediated epithelial-mesenchymal transition was induced in the newly regenerated tissue. Genes for several proteins such as sialidases and neurotrophins were identified amongst the differentially expressed transcripts. The regeneration of the ventral nerve cord was also accompanied by the induction of nerve growth factor and neurofilament genes. We identified 315 novel differentially expressed transcripts in the transcriptome, that have no homolog in any other species. Surprisingly, 82% of these novel differentially expressed transcripts showed poor potential for coding proteins, suggesting that novel ncRNAs may play a critical role in regeneration of earthworm.

## Introduction

Members of the phylum Annelida, commonly represented by earthworms and leeches are cosmopolitan in distribution, occupying a variety of niches from the soil in our gardens to marine sediments. Some species are ecologically relevant because they sieve our soil[1], while others burrow through deep marine sediments[2]. Several annelids parasitize a variety of marine and terrestrial hosts[3]. Annelids have also been found as fossilized remains from the Cambrian era[4]. Broadly divided into two major classes; Clitellata which is further divided into Oligocheates (earthworms) and Hirudinea (leeches) and the class Polychaeta (largely comprising marine worms), the systematics of this group of segmented worms undergoes constant reworking in the light of modern molecular tools employed for classification[5,6]. They belong to the superphylum Lophotrochozoa, which encompass phyla including the molluscs and flatworms, grouped according to protein coding surveys into a 402-ortholog dataset[7]. Earthworms also show intriguing behaviors like the ability to distinguish light of different wavelengths[6,8] and respond to tactile stimuli[9], vibrations[10] and dragging objects along directions that offer least resistance[11].

Many earthworm species have a remarkable ability to regenerate part of the body lost due to injury. The potential to regenerate lost body parts has repeatedly appeared and disappeared during evolution[12]. Planaria and Hydra display a type of regeneration called morphallaxis involving the reorganization of existing cells and re-programming of cell fates. The lizard tail, starfish arms and legs of some arthropods show autotomy as a defense. The breaking off of a body part is followed by re-growth and sometimes, incomplete re-establishment of cell types. The zebrafish fin and beaks of certain birds display spatially restricted regenerative potential of certain tissue types. In contrast, the earthworm and salamander limb offer models that display the coordinated regeneration of nerve, muscle, vasculature and blood.

In the presence of model organisms such as Hydra[13] or planarians[14] for studying regeneration, annelids pose an interesting new challenge. Annelids are highly variable in their regenerative capacity, with leeches showing none to some sabellids, chaetopterids and lumbriculids producing an entire individual from a mid-body segment [15]. All this hints toward a diversity of mechanisms for regeneration and asexual reproduction in annelids.

Previous attempts at characterizing the regenerative potential of earthworms have focused on the extent to which regeneration may be accomplished. Barring characterization of a few specific genes, there have been no comprehensive analyses of gene expression during regeneration in earthworms. Transcriptomic studies in the regenerating zebrafish fin[16,17], planaria[18,19] and hydra[20] have revealed the importance of growth factor signaling and re-programming factors during regeneration. Eventually, comparative analysis of genomes and transcriptomes of closely related species with widely different regenerative potential may reveal novel genes, regulatory elements or genomic rearrangements that account for the intermittent manifestation of regenerative ability during evolution.

Here, we report the transcriptome of the epigeic vermicomposting worm *Eisenia fetida*, also known as red-worm, along with an extensive analysis of transcriptome dynamics during regeneration. We report the presence of a neurotrophin gene, with limited similarity to mammalian nerve growth factor family that is upregulated during regeneration. We show that genes known to enhance neural regeneration are induced during earthworm regeneration implying that certain conserved molecular pathways are involved in this gene expression program. Based on HOX gene analysis from a draft genome Zwarycz et. al. have previously reported that the *E*. *fetida* genome has undergone extensive duplications[21]. We used miRNA as indicators of phylogenetic history to find that like Hox genes, several miRNA families have multiple paralogs in *E*. *fetida*.

In pursuit of novel drivers of regeneration, we show that 315 earthworm genes that encode proteins with no orthologs, were differentially expressed. Amongst these novel genes, 216 showed poor coding potential while 99 transcripts can potentially code for peptides that may be important in regeneration. The dominance of non-coding transcripts during regeneration may provide an explanation for the remarkable variation in regenerative potential amongst closely related annelid species.

## Materials and methods

### DNA and RNA isolation

*Eisenia fetida* earthworms were procured from farmers engaged in vermicomposting and maintained in a culture in the laboratory at around 22^o^C with moderate humidity. No specific permissions were required for procuring earthworms since they were being sold as vermicompost. They were brought in the lab and cultured as it is. They are neither endangered species nor do they come under animals requiring ethical approval. The worms were rinsed in tap water to remove any attached soil. They were then fixed in 70% ethanol for 5 minutes. A platform was used to pin the worm from both ends in order to dissect out the gut. The body wall was then cleaned thoroughly to remove all residual soil matter. The DNA was then isolated according to a protocol adapted from Adlouni et. al[22]. Briefly, the tissue was homogenized using liquid nitrogen and dissolved in 1ml of DNA extraction buffer (100mM NaCl; 50mM EDTA; 7%Sucrose; 0.5%SDS; 100mM Tris base; pH 8.8). Fifty microlitres of Proteinase K (10mg/ml) were added and the homogenate was incubated at 65^o^C for 2 hours. The proteins were precipitated by 120uL of 8M Potassium acetate at 4^o^C. Precipitated proteins were removed by centrifugation at 10,000g while the supernatant was treated with equal volume of Phenol: Chloroform: Isoamyl alcohol on ice. The aqueous layer was recovered after a 15-minute spin at 10,000g and DNA was precipitated by equal volume of Isopropanol. DNA was centrifuged at 10,000g and desalted by repeated 70% ethanol washes. The pellet was air dried and dissolved in Tris EDTA buffer (pH 7.5).

Samples from regenerating worms were collected at four different days post amputation (dpa)- immediately following amputation (0dpa), and subsequently at 15dpa, 20dpa and 30dpa. The tissue collected at 0dpa from 60 ± 6 segments was used as reference (0C) for comparison. The regenerated tissue and the tissue adjacent to it, termed control, were collected separately. RNA was isolated using the Trizol method. Briefly, the tissues were frozen in liquid nitrogen, homogenized using a mortar and pestle in the presence of 1ml Trizol reagent and transferred to a microfuge tube. Phase separation was done by adding 200uL chloroform and centrifugation at 10,000g. The aqueous phase was collected and equal volume of Isopropanol was added to precipitate RNA. The pellet was collected by centrifugation at 10,000g, followed by repeated 70% ethanol washes. The RNA was then air-dried and dissolved in nuclease-free water.

### DNA and RNA sequencing

Approximately 5ug of DNA, taken from three different worms, was sheared using Covaris S220 platform and desired fragment sizes were selected by agarose gel electrophoresis. Paired end libraries of fragment sizes 200bps and 500bps were constructed using Illumina TruSeq DNA Library Prep Kit, while three mate-pair libraries were constructed of insert sizes 10Kb, 7Kb and 5Kb (average sizes since a range was cut from the agarose gel) using Nextra Mate-Pair Library Prep Kit according to manufacturer's protocol. It should be noted that for the mate-pair library construction, shearing was performed after circularization, as per manufacturer's guidelines. Briefly, the sheared DNA was end-repaired and purified using AMPure XP beads (Beckman Coulter). This end repaired DNA was then A-tailed and ligated to adapters. The ligated DNA fragments were then amplified by PCR and purified again using AMPure XP beads generating paired end libraries for sequencing on the Illumina Platform. The 200bp-insert library was sequenced using Illumina GAnalyzer II Platform while the 500bps-insert library was sequenced using HiSeq 2500. For generating long reads (not used in the assembly), Roche 454 libraries were constructed as per manufacturer's protocol using Shotgun sequencing approach of GS FLX+ system from Roche. Genomic DNA(1μg; as estimated using Qubit high sensitivity assay) was used for rapid library preparation, which includes DNA fragmentation by nebulization, fragment end repair, adaptor ligation and small fragment removal by AMPure beads based purification. Library was quantified using Quantifluor (Promega) and qualitatively assessed by Bioanalyzer (High sensitivity chip from Agilent). The average fragment length of library was between 1400-1800 bps with <10% of total fragments under 650 bps. This was used to make working aliquots (1 x 10^7^ molecules/μl) for emulsion PCR (emPCR) standardization. For clonal amplification of the library, the optimal amount of DNA titrated using small volume emPCR was used for sequencing. After emPCR optimization, eight DNA copies per bead (cpb) were used for large volume emPCR. The enriched beads were sequenced using pyrosequencing on a picotiter plate. The raw data comprising of series of images were extracted, normalized and converted into flowgrams. The flowgrams were used during signal processing to generate analysis ready sequencing reads. The libraries were sequenced in two-region format of the picotiter plate. Lastly, for Oxford Nanopore sequencing, MAP06 kit was used according to manufacturer's protocol. Briefly, the DNA was sheared using Covaris g-Tube at 3000rpm, end repaired and A-tailed. Then, ligation was done with hairpin adapters linked to biotin labeled motor protein. The library was purified using Dynabeads M-280 and then sequenced. Metrichor was used for basecalling. Poretools was used to get FASTA sequences[23].

Approximately, 1ug of RNA was taken per sample and RNA sequencing libraries were made using TruSeq v2 Library Prep Kit as per manufacturer's protocol. Briefly, the RNA was polyA selected using OligodT magnetic beads followed by shearing into 200-500bp fragments. This sheared RNA was then used to generate cDNA. The cDNA was end-repaired to blunt ends. These blunt ends were then A-tailed i.e. an "A" overhang was added so as to ligate the adapters in the next step. The adapter-ligated cDNA was then amplified by PCR and purified by AMPure XP beads. This library was then quality checked and sequenced on Illumina HiSeq 2500. For small RNA sequencing, total RNA was isolated from body wall and sequenced on the Illumina platform. ~800ng of Total RNA was used as the starting material. Briefly, 3’ adaptors were ligated to the specific 3’OH group of microRNAs followed by 5’ adaptor ligation. The ligated products were reverse transcribed with Superscript III Reverse transcriptase by priming with Reverse transcriptase primers. The cDNA was enriched and bar-coded by PCR (15 cycles) and cleaned using Polyacrylamide gel. The library was size selected in the range of 140 – 160bp followed by overnight gel elution and salt precipitation using Glycogen, 3M Sodium acetate and absolute ethanol. The precipitate was re-suspended in Nuclease free water. The prepared library was quantified using Qubit Fluorometer, and validated for quality on High Sensitivity Bioanalyzer Chip (Agilent).

### Genome assembly

The quality of sequencing data was checked using FastQC and reads of Phred score>33 were used for further. Before starting the assembly, the quality check of the reads was done using FASTQC. The reads were trimmed to remove low quality reads (Phred score 33) using Trimmomatic. Further, reads were filtered for microbial contamination by removing reads matching any organism using an in-house database. The draft genome was made using CLC Genomics Workbench with a word size of 64 and bubble size of 50. The paired end data (obtained from Illumina HiSeq 2500 with an insert size of 500bps) was used for assembling contigs while mate pair data (with an insert size of 3.5-5.5Kb) was used for scaffolding the contigs for the final assembly. The resulting assembly was assessed using Assemblathon script to get the N50 statistics.

### Transcriptome analysis and annotation

The quality of RNA sequencing reads were checked using FastQC and reads of Phred score>33 were used for adapter trimming by Trimmomatic-v0.36 (default parameters). The data was then *de novo* assembled using Trinity-v2.3.2 package [24]. The assemblies were annotated using Transdecoder-v4.1 (http://transdecoder.sf.net) for finding ORFs. The peptides were compared to Uniprot entries using default parameters in BLAST. The samples were assembled together in a single assembly using Trinity-v2.3.2 and annotated by Trinotate. This assembly was then used as a reference to align reads of individual samples using Bowtie-v2.2.9 [25] and was then assembled using Cufflinks-v2.2.1. Read counts were obtained from alignment files using HTSeq[26]. Differential expression profiles were generated using DESeq2[27] with multiple sample correction by Benjamini Hochberg method. The data generated was then analyzed using the MATLAB suite. Small RNA sequencing data was analyzed using miRminer[28,29] pipeline. Functional classification of differentially expressed genes was carried out using DAVID. Comparisons were done against user-defined background gene list of Uniprot IDs of *E*. *fetida* orthologs. Benjamini-Hochberg corrected p-value <10^-4^ was used to select over-represented GO terms. Coding potential of transcripts was calculated using CPAT[30] and CPC2[31].

### RT PCR

The cDNA was prepared using NEB MMULV-RT enzyme using random hexamers. RT PCR was performed using Takara SYBRII in Roche LightCycler 480. The primers used have been listed in Supplementary Table (S1 Table). The products were visualized by agarose gel electrophoresis.

### PCR cloning and sanger sequencing

The cDNA was prepared using Transcriptor High Fidelity cDNA synthesis kit (Roche). The cDNA was used for PCR reaction using Fermentas Taq Polymerase. The PCR product was cloned in pET23A and sequenced using T7 primers in ABI 3130xl Genetic Analyzer according to manufacturer's protocol.

### Cryosectioning and histology

Earthworm with regenerated tails were fixed in 4% para-formaldehyde solution at room temperature for four to five hours. After fixation, the samples were washed two to three times with phosphate buffered saline (pH 7.0) at room temperature. After, washing, the samples were kept in a 15% sucrose solution (in PBS) and later in 30% sucrose solution at 4°C until the tissue was immersed. The regenerated portion of earthworm was then cut into appropriate sizes to be mounted into cryostat. The samples were frozen at -20°C in the presence of Tissue Freezing Medium (Jung). Cross-sections of 20μm thickness were cut from the regenerated portion and immediately transferred onto glass slides coated with gelatin by an artist brush and thaw mounted. The images were taken using a standard bright field microscope at 5X and 10X magnifications after hematoxylin and eosin staining.

### In situ hybridization

Regenerated earthworms (15dpa, 20dpa and 30dpa) were collected and washed with autoclaved ultrapure type-1 water, followed by overnight fixation at 4°C in 4% (w/v) para-formaldehyde (Sigma-Aldrich) prepared in 1XPBS, pH 7.4. Stringent washes using 0.1% tween-20 in 1XPBS were given to the worms with subsequent storage in 100% methanol at -20°C.

The amplified sequence of SOX4 using specific primers (S1) was cloned in TOPO2 dual promoter vector (Invitrogen). HindIII and EcoRV (New England Biolabs) were used to linearize the cloned plasmid followed by in vitro synthesis of riboprobes using digoxigenin labeled UTPs and RNA polymerase (T7 and SP6) as per the manufacture’s guidelines (Roche). Prior to hybridization, the stored worms were rehydrated in a gradient of 75%, 50%, 25% and 0% (v/v) of methanol in 0.1% tween-20 in 1XPBS. The worms were permeabilized by treatment with 20μg/ml Proteinase K for 45 minutes with subsequent fixation in 4% (w/v) PFA. Hybridization of Earthworms using the sense and antisense probes was performed at 65°C in Hybridization Buffer (50% formamide, 1.3XSSC, 5mM EDTA, 0.2% tween-20, 100μg/ml heparin and 50μg/ml yeast RNA in autoclaved ultrapure type-1 water) followed by stringent washing with TBST solution (0.5M NaCl, 0.1M KCl, 0.1M Tris-HCl (pH 7.5) and 0.1% Tween-20 in autoclaved ultrapure type-1 water). The earthworms were then incubated in 1:2000 dilution of digoxigenin-alkaline phosphatase Fab fragments (Roche) in TBST and 10% FBS at room temperature for 4 hours. The earthworms were washed with TBST and allowed to stain using 100mg/ml Nitro-blue tetrazolium (NBT) and 50 mg/ml 5-bromo-4-chloro-3ʹ-indolphosphate (BCIP) substrate (Roche) in developing solution (0.1M NaCl, 0.1M Tris-HCl (pH 9.5), 0.05M MgCl_2_, 1% tween-20 in autoclaved ultrapure type-1 water). The reaction was terminated after development of signal by washing earthworms with PBS followed by washing with stop solution (150mM NaCl, 1.2mM EDTA and 50mM MgCl_2_.6H_2_O in autoclaved ultrapure type-1 water with pH adjusted to 7.4). For image acquisition, earthworms were mounted in 2.5% methylcellulose and imaged under Nikon SMZ800N stereomicroscope at 8X, 6X, 4X and 2 X magnifications.

## Results

### Regeneration in *E*. *fetida*

Mature *E*. *fetida* with 100±10 body segments show a remarkable potential to regenerate posterior segments following amputation. Early studies on *E. fetida[32,33]*, reviewed recently by Bely and Sikes [34] mention that the posterior can also regenerate the anterior segments. However, a more recent report by Nengwen et. al. shows that the posterior can survive and regenerate the anterior segments only when the loss is restricted to the first 7 segments[35]. This is in agreement with our own observations, which prompted us to focus on the ability of *E*. *fetida* to regenerate posterior segments. To identify genes differentially expressed during regeneration, we carried out RNA sequencing and created a temporal profile of gene expression during regeneration in *E*. *fetida*.

**Fig 1:**
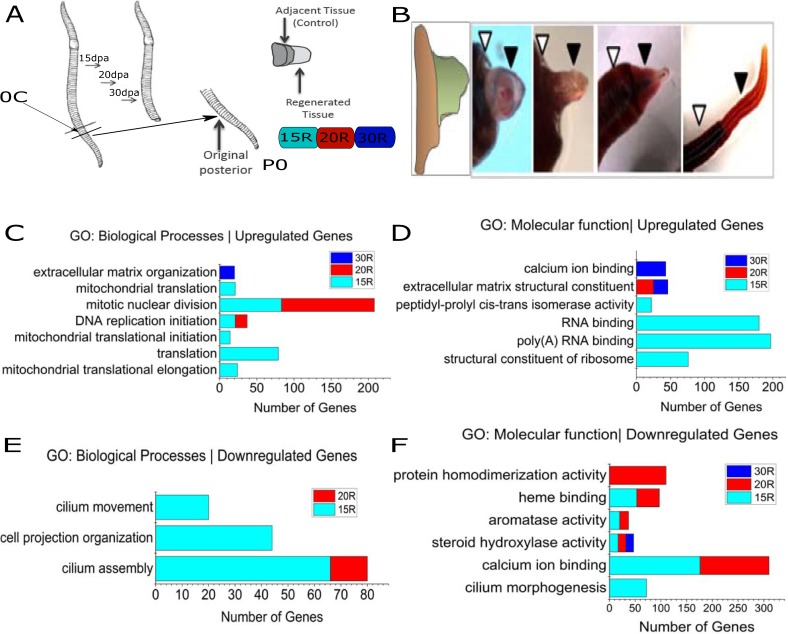
Functional classification of differentially expressed genes in the regenerating earthworm. (A) Schematic representation of the experiment: Earthworms were cut (transverse) at 60 ± 6 segments from the anterior end and anterior portion was allowed to regenerate for 15, 20 or 30 days. At 15, 20 and 30 days post amputation (dpa), the regenerated tissue and the (old) adjacent control tissue was collected. Total RNA was used for RNA-Seq analysis. (B) A typical worm during various stages of regeneration showing the regenerating tissue (filled arrowhead) and the adjacent tissue (open arrowhead). Gene ontology classification by DAVID revealed GO terms over-represented in the (C-D) upregulated genes and (E-F) downregulated genes (Benjamini Hochberg adjusted pVal ≤ 10^-4^).

In this study, earthworms were cut transversely at 60 ± 6 segments and allowed to regenerate for 15, 20 or 30 days (Fig 1A). At 15, 20 and 30 days post amputation (dpa), the regenerated tissue (henceforth referred to as 15R, 20R and 30R) and the (old) adjacent tissue (henceforth referred to as 15C, 20C and 30C) were collected (Fig 1B) from each batch consisting of 50-100 worms. In one batch, immediately after amputation, tissues were collected from the site (60 ± 6; henceforth called 0C) to be used as a reference for comparing the regenerated and control tissues. Besides these samples, we also carried out RNA-Seq of the posterior (segments 60-100; called P0 for Posterior at 0days) as shown in Fig 1A. Direct comparison of the regenerating tissue with P0 allowed us to establish the extent to which regeneration is completed by 30dpa. Three biological replicates, starting with different batches of earthworms were performed. Total RNA was used for RNA-Seq analysis and the reads were assembled, *de novo*, using Trinity [24] and annotated (see Methods). As shown in S1 Fig, comparative analysis was performed using DESeq2[27] to identify genes that were differentially expressed in any of the tissues (15C, 20C, 30C, 15R, 20R or 30R) when compared to the reference (0C). In the tissue immediately anterior to the site of injury, less than hundred genes (Table 1) were significantly differentially expressed at any point during regeneration. Gene Ontology classification using DAVID also failed to cluster these genes into any specific functional category (Table 2) on the basis of biological process or molecular function (Benjamini Hochberg corrected pVal < 10^-4^). In contrast, in the regenerating tissue, thousands of transcripts from a total of 71,341 transcripts (including isoforms) were differentially expressed (Table 1). The largest number of differentially expressed genes was in the regenerated region at 15dpa. The number of differentially expressed genes steadily reduced as regeneration proceeds, at later time points (20dpa and 30dpa). Next, we identified biological processes and molecular functions that were over-represented in the up-regulated or down-regulated genes (Fig 1C, 1D, 1E and 1F). Overall, it is clear that in the early stages of regeneration, genes involved in translation, DNA replication and cell division are upregulated while in later stages, extra-cellular matrix remodeling is undertaken.

**Table 1:** Differentially expressed genes from DESeq2 analysis.

Sample name	Number of Differentially Expressed Genes (n = 3; Adjusted pVal<0.05)	Number of Upregulated Genes (n = 3; Adjusted pVal<0.05)	Number of Downregulated Genes (n = 3; Adjusted pVal<0.05)
Segments 60 ± 6 at 0dpa	Reference	Reference	Reference
Control at 15dpa (15C)	75	64	11
Control at 20dpa (20C)	72	64	8
Control at 30dpa (30C)	63	39	24
Regenerated at 15dpa (15R)	8713	3589	5124
Regenerated at 20dpa (20R)	5499	1887	3612
Regenerated at 30dpa (30R)	1231	617	614
Posterior segments 60-100 (P0) at 0dpa	21	12	9

**Table 2:** Gene Ontology classification of differentially expressed genes.

		DESeq2	
Sample name		UP	DN
Control at 15dpa(15C), 20dpa(20C), 30dpa(30C)		“n.s”	“n.s”
Regenerated at 15dpa (15R)	BP	Mitochondrial translational elongation(24),	Cilium assembly(66),
		Translation(79),	Cell projection organization(44),
		Mitochondrial translational initiation(14),	Cilium movement(20)
		DNA replication initiation(21),	
		Mitotic nuclear division(83),	
		Mitochondrial translation(21)	
	MF	Structural constituent of ribosome(76),	Cilium morphogenesis(72),
		Poly(a) rna binding(197),	Calcium ion binding(176),
		RNA binding(180),	Steroid hydroxylase activity(17),
		Peptidyl-prolyl cis-trans isomerase activity(22)	Aromatase activity(20),
			Heme binding(53)
Regenerated at 20dpa (20R)	BP	Cell division(72),	Axoneme assembly(14)
		DNA replication initiation(16),	
		Mitotic nuclear division(53)	
	MF	Extracellular matrix structural constituent(25)	Protein homodimerization activity(110),
			Calcium ion binding(134),
			Steroid hydroxylase activity(15),
			Heme binding(44),
			Aromatase activity(17)
Regenerated at 30dpa (30R)	BP	Extracellular matrix structural constituent(21),	"n.s"
		Calcium ion binding(43)	
	MF	Extracellular matrix organization(20)	Steroid hydroxylase activity(8),
			Oxidoreductase activity(7)

Only GO categories with Benjamini Hochberg adjusted pVal<10^-4^ have been included. No significant classes found ="n.s"; Number of genes in parantheses. BP = Biological Process; MF = Molecular Function

**Fig 2:**
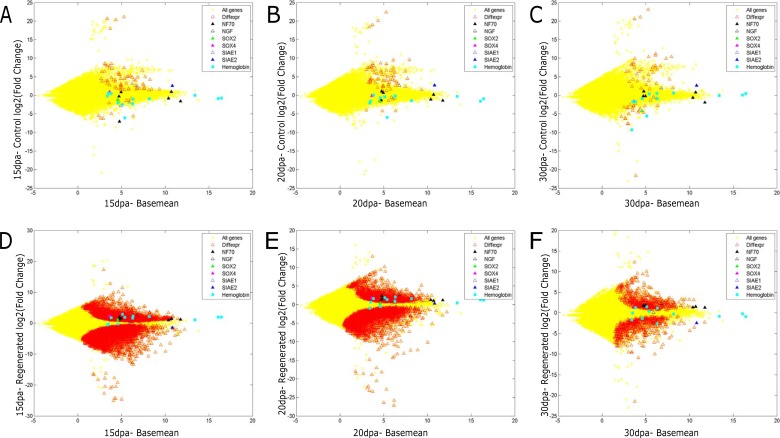
Scatter plot of fold change of genes in the (A-C) control region (Y-axis) and (D-F) regenerated region, compared to the basal expression level (basemean; X-axis) at 15, 20 and 30 days post amputation. Differentially expressed genes are marked in red. The genes analyzed in detail are highlighted. Divergent expression of two Sialidase isoforms is shown by blue triangles. Neurofilament, NF70 (black filled triangle) and Nerve Growth Factor (black open triangle) are unaffected in control tissue but induced in the regenerating tissue. Giant extra-cellular hemoglobin genes (cyan square), Sox4 (pink triangle), Sox2 (green triangle) re-gain expression in the regenerating tissue as regeneration proceeds.

We also compared the changes occurring in the regenerating tissue with the adjacent tissue. A majority of the differentially expressed genes were downregulated in the regenerating tissue when compared to the adjacent tissue (Fig 2). This apparent downregulation of genes maybe a reflection of reduced heterogeneity amongst the undifferentiated, rapidly proliferating cells in the regenerating tissue. The regenerating tissue, initially, is a homogenous mass of undifferentiated cells, wherein many genes appear downregulated because the corresponding cell type has not been formed. For instance, cilium associated genes (Fig 1E and 1F) are down regulated until the nephridia and sensory cells in the epidermis are restored. The giant extracellular hemoglobin of annelids has been studied extensively, for its exceptional oxygen carrier properties. Interestingly, the expression of the gene for giant extracellular hemoglobin (Fig 2D, 2E and 2F) of *E*. *fetida* is induced in the regenerating tissue at 15dpa and 20dpa (Adjusted pVal≤0.05). By 30dpa, the regenerated tissue has fewer differentially expressed genes, suggesting that the program of regeneration is restoring the expression of genes. Our results suggest that, following injury, the regenerating tissue mounts a well-coordinated program of gene expression.

### Regeneration factors

**Fig 3:**
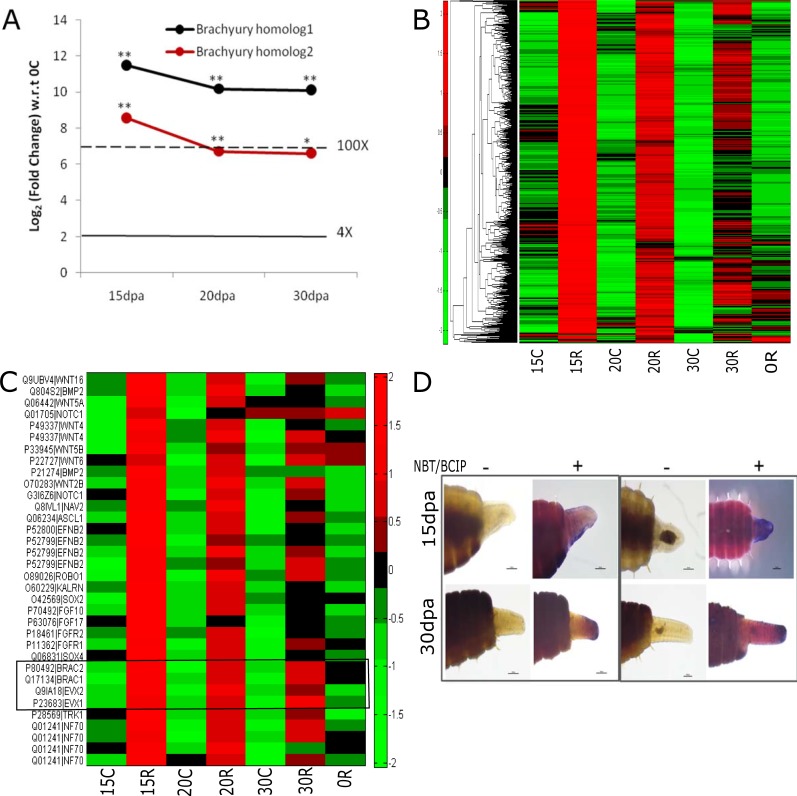
Genes induced in regenerating tissue of *E*. *fetida*. (A) Brachyury is the most highly induced gene in the regenerating tissue. The two *E*. *fetida* homologs of *Branchiostoma floridae* (lancelet) Brachyury gene are shown in black and red. * Adjusted pVal<0.05; **Adjusted pVal<0.005. (B) Cluster of 951 genes that match the profile of Brachyury in k-means clustering (C) Expression pattern of developmental genes that are highly induced in the regenerating tissue. Grey box shows Brachyury and Even-skipped. (D) SOX4 is induced in regenerating tissue at 15dpa and 30dpa. Control (probed with sense probe; left panels 1 and 2) and anti-sense probe (right panels) before (-) and after (+) addition of chromogenic agent (NBT/BCIP).

We used k-means clustering to identify co-regulated gene clusters upregulated in the regenerating tissue. The most highly induced gene (>100 fold) in the regenerating tissue is Brachyury (Fig 3A). Using the Brachyury profile as a reference, we identified a cluster of 951 genes with a strong profile match (Fig 3B). This cluster consists of several developmental regulators like FGF, BMP2, SOX4 and WNT signaling genes (Fig 3C). We verified the expression of SOX4, in the regenerating tissue using in situ hybridization. The tip of the regenerating tissue showed a strong and consistent induction of SOX4 (Fig 3D). Another gene, strongly induced in the regenerating posterior is the earthworm ortholog of the homeobox segmentation gene even-skipped (Fig 3C). In earthworm, 38 novel genes with no apparent homology in vertebrate genomes are part of the cluster of 951 genes co-expressed with Brachyury during regeneration (Fig 3B and 3C). In the absence of functional conservation, co-expression of genes can be useful in predicting function, in this instance, implicating these novel genes in regeneration and posterior development.

### Regeneration of blood and immune cells

A closer examination of the global gene expression profile, especially the genes that are strongly repressed at 15dpa revealed that a group of apparently co-regulated globins was initially depleted in the regenerating tissue. As the newly formed tissue matured, the globin genes became more abundant and eventually at 30dpa were comparable in expression to the adjacent tissue. We believe this is a reflection of the vascularization of the regenerating tissue and the associated presence of blood cells.

**Fig 4:**
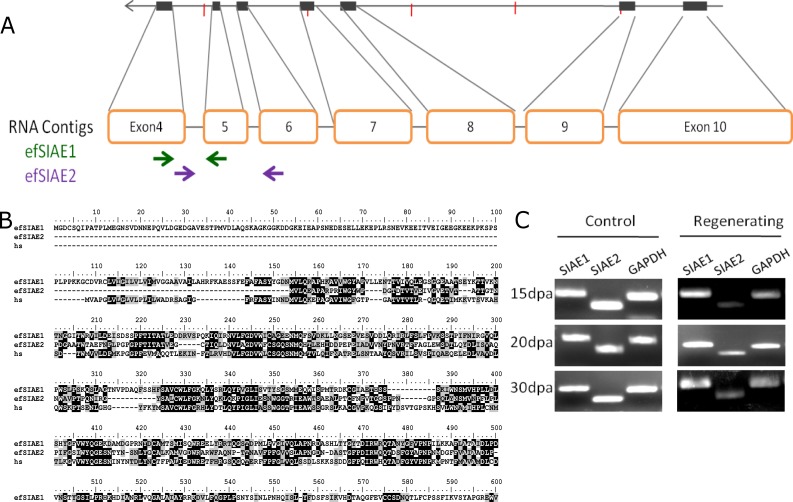
Divergent expression of O-sialic acid esterase homologs during regeneration of *E*.*fetida*: (A) Gene structure of efSIAE1. Paralog specific primers for efSIAE1 and efSIAE2 are shown by green and purple arrows respectively. (B) Two contigs derived from *de novo* assembly of RNA-Seq data showed homology at protein level to O-sialic acid esterase gene of human (hs). One of them was also identified in a scaffold assembled in the genome sequence assembly. (C) RT-PCR using gene specific primers confirmed the divergent expression of the transcripts of efSIAE1 and efSIAE2 in the RNA-Seq data.

The Sialidase (SIAE) gene, which is involved in B-cell differentiation in humans (56), had no known ortholog in the *C*. *elegans* and *Drosophila melanogaster* genomes, but genomic analysis revealed the presence of two closely related sialidase genes in the earthworm genome, occuring on two distinct contigs (Fig 4A). We were intrigued by the divergent expression patterns of the two orthologs, both of which resemble the single human SIAE gene (Fig 4B). We designed ortholog specific exon-spanning primers and validated our findings from genome assembly and RNA-Seq by RT-PCR. SIAE1, was upregulated in the regenerating tissue by 6.3 fold (data not shown) at 15days post cut while SIAE2 was downregulated (Fig 4C).

### Neural regeneration

**Fig 5:**
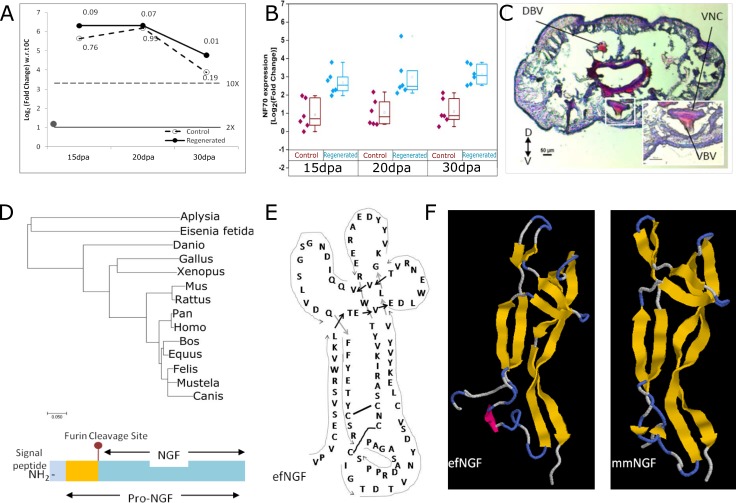
(A) Nerve Growth Factor gene is induced in regenerated tissue (solid black line) at 15dpa, 20dpa and 30dpa. Adjusted pval is mentioned at each point. The gene is also induced in the control tissue but was not statistically significant (dashed line). Stippled line shows the fold change in the posterior (P0) to the control tissue (0C) at the time of injury (0dpa). (B) Neurofilament (NF70) genes are unregulated during regeneration in *E*. *fetida*. Expression level of various isoforms of NF70 at 15, 20 and 30 days post amputation in the regenerated tissue (15R, 20R, 30R) compared to adjacent control tissue (15C, 20C, 30C). (C) Hematoxylin and Eosin (H&E) staining of cross-section of regenerating earthworm *Eisenia fetida* 20 dpa. (D) Schematic figure of NGF consisting of the signal peptide, pre-NGF and NGF separated by a conserved Furin cleavage site (sequence details in S5) with phylogenetic analysis shows that the *Eisenia fetida* does not resemble other invertebrate NGFs like the mollusc NGF in primary protein sequence. (E-F) The signal peptide and the Pro region show minimal homology but the conserved cysteines in the mature NGF reveal structural homology to mouseNGF (mmNGF).

We studied the differentially expressed genes during earthworm regeneration to identify expression profiles associated with neural regeneration in the posterior segments. The strong induction of Nerve Growth Factor and the neurofilament gene, NF70 indicated ongoing neurogenesis (Fig 5A and 5B). Earthworm anatomy shows a ventral nerve chord that connects the dorsal ganglia present in the anterior segments to the rest of the body[36,37]. In agreement with early reports[38], the ventral cord was visible in the cross-section of the regenerated tissue thus offering a model for nerve regeneration (Fig 5C). The conserved gene architecture with the presence of the characteristic furin cleavage site allowed us to align invertebrate and vertebrate NGF like genes (Fig 5D, 5E and 5F), in spite of the limited homology (S2 Fig). The annotation of the *E*. *fetida* transcriptome also comprises TrkB receptors, known to bind nerve growth factors. As shown in Fig 3C, TRK1, encoding TrkB, was amongst the highly induced genes in the regenerating tissue.

### Novel regeneration genes

Having established that the regenerating tissue of the earthworm showed differential expression of protein coding genes involved in tissue re-programming, developmental cell fate decisions and growth factor signaling, we explored the contribution of "function unknown" genes. This includes 54555 non-coding RNA containing no ORFs and 3008 transcripts which potentially code for peptides with no similarity to any known protein. Amongst 9645 differentially expressed mRNA transcripts from the DEseq2 analysis (padj<0.05), 315 had no apparent ortholog. We analyzed these genes using CPAT[30] and the more stringent CPC2[31] to verify their protein coding potential. Only 18% of the novel differentially expressed genes showed any coding potential. Of these, sixteen transcripts with no apparent ortholog or protein coding potential had a significant basal expression (>100) and were upregulated more than four fold at least at one time point during regeneration (Table 3). Taking transcript isoforms into consideration, these sixteen transcripts arise from thirteen genes. We mapped the genes to genomic scaffolds (see below) to identify protein coding genes located in the vicinity of the novel non-coding genes. One of the novel lncRNAs partially overlaps with the ABCF4 gene. Thus, the vast majority of novel regeneration genes *in E*. *fetida* were long non-coding transcripts. In spite of the annotation presented in Table 3, we cannot rule out the possibility that some of these apparently non-coding transcripts maybe fragments derived from protein coding isoforms.

**Table 3:** Novel regeneration genes from E. fetida: Selected non-coding transcripts of unknown function that were upregulated (Log_2_ Fold Change ≥2) at any timepoint during regeneration with a basal expression level (Basemean) >100. n.d = Not differentially expressed.

Trinity_ID	Expression Level (Basemean)	Fold Change (Log_2_)			Number of Isoforms	Number of Scaffolds	Nearest Known Gene Uniprot ID | Gene ID *(species*)
		15dpa	20dpa	30dpa			
DN355088_c7_g2_ i1:0-1583	142.44	3.6	2.94	2.29	1	9	Q91048|PTK7 *(Gallus gallus)* Q9JJN0|POLH (*Mus musculus*)
DN307027_c2_g1_ i1:0-895	1514.3	3.3	2.56	3	1	7	P18048|SPIK2 (*Human adenovirus 40*)
DN336427_c40_g22_i2:0-1571	505.79	3.05	2.62	n.d.	3	12	Q14767|LTBP2 (*Homo sapiens*)
DN336427_c40_g22_i1:0-1040	110.81	2.67	n.d.	n.d.	3	10	
DN336427_c40_g22_i3:0-1594	131.56	2.14	n.d.	n.d.	3	11	
DN343980_c2_g12_ i1:0-959	631.67	n.d.	n.d.	3.02	1	7	Q54HT7|ADCF (*Disctyostelium discoideum*)
DN340881_c2_g2_ i1:0-933	281.44	2.98	2.45	n.d.	1	6	Q8T6B4|ABCF4 (*Disctyostelium discoideum*)
DN351907_c1_g3_ i2:0-2004	162.40	2.93	2.74	n.d.	1	5	Q5VT52|RPRD2 (*Homo sapiens*) P56574|IDHP (*Rattus rattus*)
DN351907_c1_g3_ i3:0-1970	298.92	2.01	n.d.	n.d.	1	5	
DN345132_c3_g25_ i1:0-1411	138.20	2.78	n.d.	n.d.	2	13	Q1RMU3|P4HA1 (*Mus musculus*)
DN345132_c3_g25_ i2:0-1423	369.30	1.31	1.42	0.66	2	13	
DN355787_c5_g4_ i1:0-1141	858.38	2.63	2.66	n.d.	1	4	P98160|PGBM (*Homo sapiens*)
DN345045_c0_g1_ i1:0-1568	103.33	2.59	n.d.	n.d.	1	6	P04146|COPIA (*Drosophila melanogaster*)
DN329618_c1_g9_ i1:0-927	221.49	2.13	n.d.	n.d.	1	2	Q7ZY29|ESRP1 (*Xenopus laevis*)
DN349882_c1_g8_ i1:0-1762	1115.7	2.47	2.44	n.d.	1	9	P17140|CO4A2 (*Caenorhabditis elegans*)
DN287307_c0_g1_ i1:0-1499	125.41	2.22	n.d.	n.d.	1	7	Q54YN3|EMC3 (*Disctyostelium discoideum*)
DN355098_c0_g5_ i2:0-2055	106.52	2.01	n.d.	n.d.	2	8	Q70KP1|HEMA (*Porcine torovirus*)
DN355098_c0_g5_ i4:0-1749	25.51	1.87	1.61	1.09	2	8	

To complement the transcriptomics analyses presented above, we also characterized the miRNome of *E*. *fetida*. Small non-coding RNAs, especially microRNAs have been used as phylogenetic markers to understand evolutionary relations between closely related organisms and genome duplications during evolution. We sequenced the small RNA pool and the genomic DNA of the *E*. *fetida* body wall to identify and annotate the microRNAome of the red worm.

### *E*. *fetida* genome sequence

The diploid genome of a group of *Eisenia fetida* worms from a vermicompost culture maintained in the laboratory was sequenced. We confirmed the species of the worms by sequencing the variable region of cytochrome c oxidase amplified using universal primers recommended for DNA bar-coding [39,40]. The whole-genome shotgun sequencing and de novo assembly was based on Illumina short reads from the genome and a mate-pair library for scaffolding. The best assembly provided 4,63,133 contigs (N50 = 967bp) and 3,99,006 scaffolds (N50 = 9Kb) (Table 4). Vitturi et. al. have previously estimated the genome size, chromosome number and GC content of two members of oligochaetae: Octodrilus complanatus and *Eisenia fetida*[41]. The diploid chromosome number has been shown to be 22[42]. In good agreement with their findings, we also report that the GC content of the *E*. *fetida* genome is 40%. The genome size estimated from nuclear DNA content (1.37 X 10^9^) also agrees well with the genome size reported here (1.4 X10^9^). The scaffolds were obtained by assembling 1.3 billion paired-end Illumina reads (read length = 100bp) and 398 million reads from a mate pair library of 10Kb. The quality of the assembled genome was verified using BUSCO[43], wherein 18.2% of the 303 BUSCO groups searched were identified in our genome. By assessing the ability to detect the 4,329 EST sequences of *E*. *fetida* that were previously available in Genbank, we found 85.8% of the previously known ESTs (3,718 of 4,329) were identified in our genome assembly. We also carried out a de novo assembly of RNA-Seq reads from the body wall of a pool of *E*. *fetida* worms using the Trinity package. 94.6% (67,491 out of 71,341) of Transdecoder verified transcripts were mapped to at least one scaffold of the genome. We also carried out RNA-Seq and *de novo* assembly for tissue collected from the regenerating region earthworms during three stages of regeneration (15, 20 and 30 days post amputation). A combined assembly of all the RNA-Seq data led to the identification of 42,610 genes collectively accounting for 71,341 transcript. The mitochondrial genome sequence was assembled as a single contig of 16kb from an assembly of 1.1 million Roche 454 reads (read length = 1000bp) and showed high conservation to the mitochondrial genomes of the deep-dwelling earthworm *Lumbricus terrestris* and the marine oligochaete, *Capitella teleta* ([44,45], S3 Fig). During the course of this study, Zwarycz et. al. have also sequenced a North American isolate of *E*. *fetida*[21]. Our assembly has approximately five fold higher contig N50 and Scaffold N50 (see Table 4 for comparison of both assemblies). Zwarcyz et. al. reported, from the analysis of HOX genes, that an exceptionally large number of duplication events have occurred in the earthworm lineage[21].

**Table 4:** Genome assembly of *E*. *fetida*.

Parameter	Zwarycz et al. 2015	Illumina/CLC assembler/CLC Scaffolding with MP data
**Total sequence length**	1,052,631,503	1,472,003,768
**Total assembly gap length**	314,083,977	1,016,501,040
**Number of scaffolds**	1,659,527	399,006
**Scaffold N50**	1,852	9,314
**Scaffold L50**	141,722	46,183
**Number of contigs**	4,728,942	463,133
**Contig N50**	199	967
**Contig L50**	917,147	132,943
**Total length (Mb)**	1052.63	1472
**%GC**	28.6	40

### miRNome: Genome duplications

**Fig 6:**
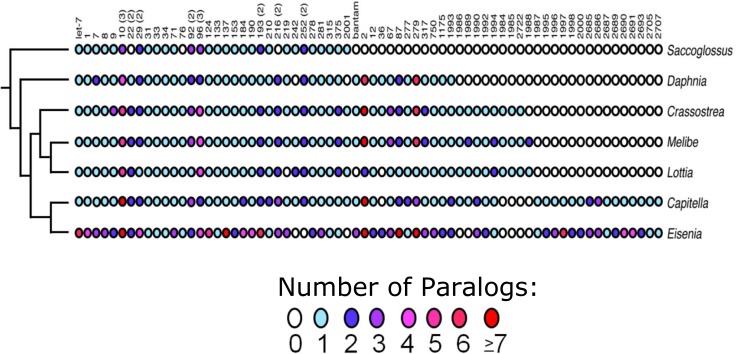
The acquisitional history of 68 microRNA families in select invertebrate bilaterians with well curated microRNAomes. Also shown is the number of microRNA paralogs per family in each taxon. Some families were tandemly duplicated early in bilaterian history including mir-10, mir-22, mir-29 and mir-96; the number of reconstructed genes for each family in the bilaterian last common ancestor is shown in parentheses. Most incidences of paralogy involve these families in these (and most other invertebrate) taxa. *Eisenia* is a notable exception in that most families are characterized by the possession of at least two paralogs, and many families have many more, consistent with the hypothesis that a (or multiple) genome duplication events occurred in this lineage after the split from the polychaete *Capitella*. See S6 for further details.

Using MiRminer[28,29] we identified 190 microRNA genes belonging to 66 miRNA families, including 18 gene families present only in other lophotrochozoans, neotrochozoans, or annelid worms, and 7 novel miRNA genes not found in *Capitella teleta* or any other metazoan thus far investigated (S2 Table; Fig 6). Like most metazoan taxa[46,47], the amount of miRNA family loss appears to be small; these losses, affecting five different miRNA families where neither a locus was detected in the various assemblies nor reads detected in our small RNA library (S2 Table), appear to be shared with *Lumbricus terrestrialis*[29], and thus most likely occurred after oligochaetes split from *C*. *teleta*, but before the split between the two earthworm species.

In contrast to *C*. *teleta* or any other invertebrate yet investigated, *E*. *fetida* is rich in miRNA paralogs averaging nearly 2.5 paralogs per ancestral gene (S3 Table). Some families, like Mir-10, Mir-124 and Mir-137, have more copies per gene family than any other animal (including human) yet investigated. Because each of these paralogs is characterized by a unique star (and often mature) sequence, and reads of the mature (and often star) sequence were detected in our small RNA library (S2 Table), the unusually high number of paralogs cannot simply be due to unrecognized heterozygosity (see Zwarycz et al. 2015[21]). Instead, our observation is consistent with what is known regarding homeobox genes in this worm; Zwarycz et al.[21] found that *E*. *fetida* has at least 364 homeobox genes with multiple representatives of *Hox* genes including four *Hox1*/*labial*, *Post1* and *Post2* genes; and at least three each of *Hox3*, *Hox5*/*Scr*, *Lox2*, and *Lox4*. Indeed, we found multiple copies of Mir-10 genes associated within the *Hox* cluster[48] including two mir-10s, three mir-993 genes, two copies of mir-10b (a mir-10 paralog found in annelids and molluscs), and one copy each of mir-1991 and mir-10c (an annelid-specific mir-10 paralog) (S2 Table). Therefore, the microRNAs we found in this worm are consistent with the idea that large, if not whole-scale, gene/genome duplication events occurred in this lineage after its split from *C. teleta[21]*, duplications that affected the entire genome including the miRNAs.

## Discussion

Studies in comparative regeneration within annelids would provide insight into the gain or loss of regenerative capability over a short evolutionary span. Some species that recently lost regenerative capability might possess a latent ability suppressed due to evolutionary loss of key genes[15]. In Hydra and some vertebrates regeneration has been ‘rescued’ by experimental expression of certain vital signaling molecules, Wnt-signalling pathway members [49,50], membrane proteins [51] or by suppression of immune responses [52]. In annelids, work on marine naidine annelids has yielded that the position and developmental stage of amputations made, can ‘naturally’ rescue regenerative capacities[34]. The ability to regenerate anterior segments has been lost at least 12 times while the posterior regenerative ability was lost at least 4 times during the evolution of annelids [53]. The genome of *E*. *fetida* can in future provide the basis for comparative genomics of regenerating and non-regenerating species within the large class of annelids.

Regenerating regions of earthworm showed a robust induction of the brachyury gene. Brachyury, literally meaning short-tail after the heritable shortened tail of mice that carry a mutation in this transcription factor[54,55], is expressed in the mesoderm and is required for the specification of posterior structures in fruit flies[56], sea urchins[57], nematodes[58], zebrafish[59], frogs[60], chicken[61] and humans, suggesting a conserved role across evolution [62,63]. In the hydra and newt, it has been shown to be induced in regenerating tissues [64,65]. In agreement with our RNAseq results, in situ hybridization studies in *Capitella telata*[66] and *Platynereis dumerilii*[67] have shown that even skipped is a marker of posterior development. Further, it has been implicated in zebrafish fin regeneration [68]. Taken together, the similarities between the genes induced in the regenerating regions, suggest a common regeneration program in these organisms.

SOX4 was also co regulated with Brachyury in the regenerating tissue. Sox4 is known to be a master regulator of epithelial to mesenchymal transition[69]. Our Gene Ontology analysis had shown that ECM genes were over-represented amongst the genes upregulated in the regenerating tissue. In earthworm, we found that SOX4 was strongly induced at the distal tip of the regenerating tissue. Taken together, the induction of SOX4, a master regulator of EMT and ECM genes suggests that signaling through the extra-cellular matrix is a vital component of regeneration in *E*. *fetida*.

Subtractive hybridization experiments in regenerating fragments of *Enchytraeus japonensis* revealed that novel genes (which are not represented in other phyla or even the *Lumbricus rubellus*) were upregulated in regenerative processes hinting towards the involvement of annelid specific genes in regeneration [70]. Gene expression profiling in anterior regenerating segments of *Perionyx excavatus* also yielded several novel ESTs, involved in regeneration[71]. Transcriptomics analysis of a mixed-stage sample of regeneration and fission in *Pristina leidyi* has also been recently attempted [72]. In our study, we found that 315 amongst the 9,645 genes upregulated during regeneration had no known ortholog. The large fraction of genes of unknown function suggests that, like in other annelid species, novel factors are involved in *E*. *fetida* regeneration. Besides these novel factors, well known re-programming genes like SOX4 [73] and Brachyury [64,74] were also induced in the regenerating tissue. The co-regulation of these and the invertebrate specific factors (conserved differentiation factors) implies that they work in unison. We speculate that novel factors derived from earthworms could participate in cellular networks driven by mammalian reprogramming factors. For instance, our analyses revealed the presence of an earthworm Nerve Growth Factor with only rudimentary similarities to mammalian counterparts, but the Trk receptors showed a higher degree of conservation. In future, functional regeneration assays in mammalian nerve injury models using earthworm-derived factors can ascertain the barriers to regeneration in the mammalian nervous system.

Neural regeneration is diminished even in tail regeneration models like the leopard gecko, *E*. *macularius*, which shows robust regeneration of a hollow surrounding cartilaginous cone devoid of the spinal cord within [75]. Neurofilament, a major component of the neuronal cytoskeleton are primarily responsible for providing structural support for the axon and regulating axon diameter [76,77]. Mammalian neural regeneration is accompanied by the tight regulation and time dependent differential expression of neurofilament genes[78]. Besides neurofilament, nerve growth factors play an important role in neurogenesis, axon guidance[79] and appropriate innervations of regenerated tissue following injury in vertebrates. Nerve growth factors were not known in invertebrates and were even contested until recently. The presence of a nerve growth factor in *Aplysia* was thought to be a unique feature of molluscs amongst invertebrates [80]. The regenerative ability of *E*. *fetida* prompted us to explore the newly sequenced genome for NGF-like genes. We report here, the identification of a 747bp pro-NGF, which can give rise to a 681bp mature form with limited similarity to mammalian NGF. The regenerated ventral nerve cord was evident in histology while the poorly conserved NGF gene and neurofilament genes were induced in the regenerating earthworm. Taken together, it is clear that the neural tissue in earthworm is restored after regeneration.

Sialic acids are the N- or O-substituted derivatives of the 9-carbon monosaccharide[81], neuraminic acid, N-acetylneuraminic acid being the predominant form in mammalian neural cells while O-acetyl neuraminic acid is a marker for differentiation of immune cells[82,83]. In mammalian genomes, lysosomal and cytosolic isoforms of sialic acid 9-O-acetylesterase catalyze the removal of 9-O-acetylation and play an important role in auto-immunity and B-cell differentiation [84–86]. Earthworms, especially *E*. *fetida* have been studied extensively as a model for invertebrate innate immunity [87], but the role of SIAE has never been reported. Sialidase (SIAE) showed two paralogs with divergent expression pattern at all time points in the regenerated tissue, compared to the adjacent tissue. The expansion of Hox genes, miRNA genes and the SIAE genes suggest that gene duplication is a recurrent phenomenon in the evolutionary history of *E*. *fetida*. While the impact of this gene duplication and divergent regulation is yet unknown, the knowledge of novel orthologs may provide deeper insights into aspects of sialic acid mediated immune cell differentiation.

Varhalmi et. al. have previously studied the expression of orthologs of pituitary adenylate cyclase-activating polypeptide (PACAP) using radioimmunoassay and immunohistochemistry in *E*. *fetida* [88,89]. Since these factors are Neurotrophin in vertebrates, it was thought that their accumulation in the regenerating tissue of *E*. *fetida* was critical for its innervation. However, the sequences of these factors were not known and the authors relied on immunoreactivity to antibodies against orthologous human proteins. In our experiments, at 15dpa and 20dpa, the earthworm ortholog of PACAP type I receptor was downregulated in the regenerating tissue.

*Eisenia fetida*, or the red worm, is a surface dwelling epigeic annelid, distributed on every continent except Antarctica. Genetic mapping and profiling exercises have involved microsatellite markers[90–92] and AFLP[93]. Attempts have been made at bar-coding species using mitochondrial DNA markers such as COI and COII [93,94]. Terrestrial annelids are now represented by the genome of *E*. *fetida* (Indian isolate), reported here and an independently sequenced North American strain [21]. The ongoing *Lumbricus* genome sequencing effort is also likely to complement information derived from the *E*. *fetida* genomes paving the way for a detailed comparative analysis of deep burrowing and surface dwelling worms. For instance, the genome of the highly photophobic *E*. *fetida* contains several opsin-like sequences. Comparative genomics between deep dwelling and surface dwelling worms in the distribution and wavelength spectra of opsins could reveal interesting insights into molecular basis of behavior.

The emergence of affordable, next generation sequencing technology has enabled comparative genomics of vertebrates, but sequences of invertebrate genomes, especially larger ones has remained scant. By sequencing the genome along with a detailed transcriptomics analysis, we were able to identify novel factors involved in invertebrate specific regeneration. Study of invertebrate biology has in the past led to unforeseen applications of human use exemplified by the discovery of Green Fluorescent protein from *Aequorea victoria* that has led to an array of technologies. The earthworm genome and metagenome holds promise of novel factors involved in regeneration and a better understanding of soil ecology, paving the way for genomics based tools to study genetic diversity. The large diversity of earthworm species suggests that the *E*. *fetida* genomes can serve as a node for cross-species genome-scale comparisons.

## Supporting information

S1 Table Primers used in this study. (DOCX)

S2 Table miRNAs annotated from *E*. *fetida* small RNA transcriptome and genome. (XLSX)

S3 Table Analysis of paralogs in *E*. *fetida* miRNome. (XLSX)

S1 Fig Flowchart of gene annotation and differential expression analysis. (EPS)

S2 Fig Annotation of nerve growth factor from *E*. *fetida*. (DOCX)

S3 Fig The mitochondrial genome of *E*. *fetida*. (EPS)

## References

[1] Drake HL, Horn MA (2007) As the worm turns: the earthworm gut as a transient habitat for soil microbial biomes. Annu Rev Microbiol 61: 169–189. 10.1146/annurev.micro.61.080706.093139 17506687

[2] Kristensen E, Kostka J (2005) Macrofaunal burrows and irrigation in marine sediment: microbiological and biogeochemical interactions. Interactions between macro-and microorganisms in marine sediments: 125–157.

[3] Uebelacker JM (1978) A new parasitic polychaetous annelid (Arabellidae) from the Bahamas. The Journal of Parasitology: 151–154.

[4] Parry L, Vinther J, Edgecombe GD (2015) Cambrian stem-group annelids and a metameric origin of the annelid head. Biology Letters 11. 10.1098/rsbl.2015.0763 PMC4650189 26445984

[5] Brusca RC, Brusca GJ, Haver NJ (1990) Invertebrates: Sinauer Associates Sunderland, Massachusetts.

[6] Edwards CA, Bohlen PJ (1996) Biology and ecology of earthworms: Springer Science & Business Media.

[7] Laumer CE, Bekkouche N, Kerbl A, Goetz F, Neves RC, et al (2015) Spiralian phylogeny informs the evolution of microscopic lineages. Curr Biol 25: 2000–2006. 10.1016/j.cub.2015.06.068 26212884

[8] Merker E, Braunig G (1927) Die Empfindlichkeit feucchthautiger Tiere im Lichte. 3. Die Empfindlichkeit feucchthautiger Tiere im Licht der Quarzquecksiblerlampe. Zool Jb Abt Allgem Zool Physiol, Tiere 43: 275–338.

[9] Moore M (1979) The rapid escape response of the earthworm Lumbricus terrestris L.: overlapping sensory fields of the median and lateral giant fibres. The Journal of Experimental Biology 83: 231–238.

[10] Mitra O, Callaham MA Jr., Smith ML, Yack JE (2009) Grunting for worms: seismic vibrations cause Diplocardia earthworms to emerge from the soil. Biol Lett 5: 16–19. 10.1098/rsbl.2008.0456 18854292 PMC2657739

[11] Wilson WJ, Ferrara NC, Blaker AL, Giddings CE (2014) Escape and avoidance learning in the earthworm Eisenia hortensis. PeerJ 2: e250 10.7717/peerj.250 24498578 PMC3912444

[12] Ozpolat BD, Bely AE (2016) Developmental and molecular biology of annelid regeneration: a comparative review of recent studies. Curr Opin Genet Dev 40: 144–153. 10.1016/j.gde.2016.07.010 27505269

[13] Galliot B, Chera S (2010) The Hydra model: disclosing an apoptosis-driven generator of Wnt-based regeneration. Trends Cell Biol 20: 514–523. 10.1016/j.tcb.2010.05.006 20691596

[14] Dalyell JG (2007) Observations on some interesting phenomena in animal physiology, exhibited by several species of planariae: Dyer Press.

[15] Bely AE (2006) Distribution of segment regeneration ability in the Annelida. Integrative and Comparative Biology 46: 508–518. 10.1093/icb/icj051 21672762

[16] Nachtrab G, Kikuchi K, Tornini VA, Poss KD (2013) Transcriptional components of anteroposterior positional information during zebrafish fin regeneration. Development 140: 3754–3764. 10.1242/dev.098798 23924636 PMC3754474

[17] Rabinowitz JS, Robitaille AM, Wang Y, Ray CA, Thummel R, et al (2017) Transcriptomic, proteomic, and metabolomic landscape of positional memory in the caudal fin of zebrafish. Proc Natl Acad Sci U S A 114: E717–E726. 10.1073/pnas.1620755114 28096348 PMC5293114

[18] Sandmann T, Vogg MC, Owlarn S, Boutros M, Bartscherer K (2011) The head-regeneration transcriptome of the planarian Schmidtea mediterranea. Genome Biol 12: R76 10.1186/gb-2011-12-8-r76 21846378 PMC3245616

[19] Lapan SW, Reddien PW (2012) Transcriptome analysis of the planarian eye identifies ovo as a specific regulator of eye regeneration. Cell Rep 2: 294–307. 10.1016/j.celrep.2012.06.018 22884275 PMC3785364

[20] Petersen HO, Hoger SK, Looso M, Lengfeld T, Kuhn A, et al (2015) A Comprehensive Transcriptomic and Proteomic Analysis of Hydra Head Regeneration. Mol Biol Evol 32: 1928–1947. 10.1093/molbev/msv079 25841488 PMC4833066

[21] Zwarycz AS, Nossa CW, Putnam NH, Ryan JF (2015) Timing and scope of genomic expansion within Annelida: evidence from homeoboxes in the genome of the earthworm Eisenia fetida. Genome biology and evolution: evv243. 10.1093/gbe/evv243 PMC4758240 26659921

[22] el Adlouni C, Mukhopadhyay MJ, Walsh P, Poirier GG, Nadeau D (1995) Isolation of genomic DNA from the earthworm species Eisenia fetida. Mol Cell Biochem 142: 19–23. 7753038 10.1007/BF00928909

[23] Loman NJ, Quinlan AR (2014) Poretools: a toolkit for analyzing nanopore sequence data. Bioinformatics 30: 3399–3401. 10.1093/bioinformatics/btu555 25143291 PMC4296151

[24] Grabherr MG, Haas BJ, Yassour M, Levin JZ, Thompson DA, et al (2011) Full-length transcriptome assembly from RNA-Seq data without a reference genome. Nat Biotechnol 29: 644–652. 10.1038/nbt.1883 21572440 PMC3571712

[25] Langmead B, Trapnell C, Pop M, Salzberg SL (2009) Ultrafast and memory-efficient alignment of short DNA sequences to the human genome. Genome Biol 10: R25 10.1186/gb-2009-10-3-r25 19261174 PMC2690996

[26] Anders S, Pyl PT, Huber W (2015) HTSeq--a Python framework to work with high-throughput sequencing data. Bioinformatics 31: 166–169. 10.1093/bioinformatics/btu638 25260700 PMC4287950

[27] Love MI, Huber W, Anders S (2014) Moderated estimation of fold change and dispersion for RNA-seq data with DESeq2. Genome Biol 15: 550 10.1186/s13059-014-0550-8 25516281 PMC4302049

[28] Wheeler BM, Heimberg AM, Moy VN, Sperling EA, Holstein TW, et al (2009) The deep evolution of metazoan microRNAs. Evol Dev 11: 50–68. 10.1111/j.1525-142X.2008.00302.x 19196333

[29] EA S, J V, VN M, BM W, M S, et al (2009) - MicroRNAs resolve an apparent conflict between annelid systematics and their. Proc Biol Sci 276: 4315–4322. 10.1098/rspb.2009.1340 19755470 PMC2817109

[30] Wang L, Park HJ, Dasari S, Wang S, Kocher JP, et al (2013) CPAT: Coding-Potential Assessment Tool using an alignment-free logistic regression model. Nucleic Acids Res 41: e74 10.1093/nar/gkt006 23335781 PMC3616698

[31] Kang YJ, Yang DC, Kong L, Hou M, Meng YQ, et al (2017) CPC2: a fast and accurate coding potential calculator based on sequence intrinsic features. Nucleic Acids Res 45: W12–W16. 10.1093/nar/gkx428 28521017 PMC5793834

[32] Gates G (1949) Regeneration in an earthworm, Eisenia foetida (Savigny) 1826. I. Anterior regeneration. The Biological Bulletin 96: 129–139. 18120625

[33] Moment GB (1949) Segment frequencies in anterior regeneration in the earthworm, Eisenia foetida. J Exp Zool 111: 449–456. 18142377 10.1002/jez.1401110307

[34] Bely AE, Sikes JM (2010) Latent regeneration abilities persist following recent evolutionary loss in asexual annelids. Proc Natl Acad Sci U S A 107: 1464–1469. 10.1073/pnas.0907931107 19966282 PMC2824374

[35] Nengwen X, Feng G, Edwards CA (2011) The regeneration capacity of an earthworm, Eisenia fetida, in relation to the site of amputation along the body. Acta Ecologica Sinica (International Journal) 31: 197–204.

[36] Wallwork JA (1983) Earthworm biology: E. Arnold (Publishers) Ltd.

[37] Levi JU, Cowden RR, Collins GH (1966) The microscopic anatomy and ulrastructure of the nervous system in the earthworm (Lumbricus sp.) with emphasis on the relationship between glial cells and neurons. Journal of Comparative Neurology 127: 489–509. 10.1002/cne.901270405 4165525

[38] Painter BT (1940) The location of factors of head regeneration in the earthworm. The Biological Bulletin 78: 463–485.

[39] Folmer O, Black M, Hoeh W, Lutz R, Vrijenhoek R (1994) DNA primers for amplification of mitochondrial cytochrome c oxidase subunit I from diverse metazoan invertebrates. Mol Mar Biol Biotechnol 3: 294–299. 7881515

[40] Römbke J, Aira M, Backeljau T, Breugelmans K, Domínguez J, et al (2015) DNA barcoding of earthworms (Eisenia fetida/andrei complex) from 28 ecotoxicological test laboratories. Applied Soil Ecology. 10.1016/j.apsoil.2015.04.005

[41] Vitturi R, Colomba MS, Pirrone A, Libertini A (2000) Physical mapping of rDNA genes, (TTAGGG)n telomeric sequence and other karyological features in two earthworms of the family Lumbricidae (Annelida: Oligochaeta). Heredity (Edinb) 85 Pt 3: 203–207. 11012723 10.1046/j.1365-2540.2000.00709.x

[42] Vitturi R, Colombera D, Catalano E, Amico F (1991) Karyotype analysis, nucleolus organizer regions and C-banding pattern of Eisenia foetida (Oligochaeta, Lumbricidae). Genetica 83: 159–165.

[43] Simao FA, Waterhouse RM, Ioannidis P, Kriventseva EV, Zdobnov EM (2015) BUSCO: assessing genome assembly and annotation completeness with single-copy orthologs. Bioinformatics 31: 3210–3212. 10.1093/bioinformatics/btv351 26059717

[44] Boore JL, Brown WM (1995) Complete sequence of the mitochondrial DNA of the annelid worm Lumbricus terrestris. Genetics 141: 305–319. 8536978 10.1093/genetics/141.1.305 PMC1206728

[45] Simakov O, Marletaz F, Cho SJ, Edsinger-Gonzales E, Havlak P, et al (2013) Insights into bilaterian evolution from three spiralian genomes. Nature 493: 526–531. 10.1038/nature11696 23254933 PMC4085046

[46] B F, T B, LE P, M J, JE T, et al (2015) - A Uniform System for the Annotation of Vertebrate microRNA Genes and the. Annu Rev Genet 49: 213–242. 10.1146/annurev-genet-120213-092023 26473382 PMC4743252

[47] JE T, EA S, A N, AM H, JM R, et al (2013) - miRNAs: small genes with big potential in metazoan phylogenetics. Mol Biol Evol 30: 2369–2382. 10.1093/molbev/mst133 23913097

[48] A H E M (2012) - Building a robust a-p axis. Curr Genomics 13: 278–288. 10.2174/138920212800793348 23204917 PMC3394115

[49] Kawakami Y, Rodriguez Esteban C, Raya M, Kawakami H, Marti M, et al (2006) Wnt/beta-catenin signaling regulates vertebrate limb regeneration. Genes Dev 20: 3232–3237. 10.1101/gad.1475106 17114576 PMC1686599

[50] Lengfeld T, Watanabe H, Simakov O, Lindgens D, Gee L, et al (2009) Multiple Wnts are involved in Hydra organizer formation and regeneration. Dev Biol 330: 186–199. 10.1016/j.ydbio.2009.02.004 19217898

[51] Adams DS, Masi A, Levin M (2007) H+ pump-dependent changes in membrane voltage are an early mechanism necessary and sufficient to induce Xenopus tail regeneration. Development 134: 1323–1335. 10.1242/dev.02812 17329365

[52] Fukazawa T, Naora Y, Kunieda T, Kubo T (2009) Suppression of the immune response potentiates tadpole tail regeneration during the refractory period. Development 136: 2323–2327. 10.1242/dev.033985 19515697

[53] Martinez‐Acosta VG, Zoran MJ Evolutionary Aspects of Annelid Regeneration. eLS.

[54] Clark FH (1934) Linkage Studies of Brachyury (Short Tail) in the House Mouse. Proc Natl Acad Sci U S A 20: 276–279. 16587886 10.1073/pnas.20.5.276 PMC1076400

[55] Wu B, Shao Y, Chen B, Liu C, Xue Z, et al (2010) Identification of a novel mouse brachyury (T) allele causing a short tail mutation in mice. Cell Biochem Biophys 58: 129–135. 10.1007/s12013-010-9097-9 20809182

[56] Kispert A, Herrmann BG, Leptin M, Reuter R (1994) Homologs of the mouse Brachyury gene are involved in the specification of posterior terminal structures in Drosophila, Tribolium, and Locusta. Genes Dev 8: 2137–2150. 7958884 10.1101/gad.8.18.2137

[57] Peterson KJ, Harada Y, Cameron RA, Davidson EH (1999) Expression pattern of Brachyury and Not in the sea urchin: comparative implications for the origins of mesoderm in the basal deuterostomes. Dev Biol 207: 419–431. 10.1006/dbio.1998.9177 10068473

[58] Woollard A, Hodgkin J (2000) The caenorhabditis elegans fate-determining gene mab-9 encodes a T-box protein required to pattern the posterior hindgut. Genes Dev 14: 596–603. 10716947 PMC316422

[59] Schulte-Merker S, van Eeden FJ, Halpern ME, Kimmel CB, Nusslein-Volhard C (1994) no tail (ntl) is the zebrafish homologue of the mouse T (Brachyury) gene. Development 120: 1009–1015. 7600949 10.1242/dev.120.4.1009

[60] Benitez MS, Del Pino EM (2002) Expression of Brachyury during development of the dendrobatid frog Colostethus machalilla. Dev Dyn 225: 592–596. 10.1002/dvdy.10190 12454936

[61] Kispert A, Ortner H, Cooke J, Herrmann BG (1995) The chick Brachyury gene: developmental expression pattern and response to axial induction by localized activin. Dev Biol 168: 406–415. 10.1006/dbio.1995.1090 7729577

[62] Smith J (1999) T-box genes: what they do and how they do it. Trends Genet 15: 154–158. 10203826 10.1016/s0168-9525(99)01693-5

[63] Kavka AI, Green JB (1997) Tales of tails: Brachyury and the T-box genes. Biochim Biophys Acta 1333: F73–84. 9395282 10.1016/s0304-419x(97)00016-4

[64] Technau U, Bode HR (1999) HyBra1, a Brachyury homologue, acts during head formation in Hydra. Development 126: 999–1010. 9927600 10.1242/dev.126.5.999

[65] Sone K, Takahashi TC, Takabatake Y, Takeshima K, Takabatake T (1999) Expression of five novel T-box genes and brachyury during embryogenesis, and in developing and regenerating limbs and tails of newts. Dev Growth Differ 41: 321–333. 10400394 10.1046/j.1440-169x.1999.413435.x

[66] Seaver EC, Yamaguchi E, Richards GS, Meyer NP (2012) Expression of the pair-rule gene homologs runt, Pax3/7, even-skipped-1 and even-skipped-2 during larval and juvenile development of the polychaete annelid Capitella teleta does not support a role in segmentation. Evodevo 3: 8 10.1186/2041-9139-3-8 22510249 PMC3359188

[67] de Rosa R, Prud'homme B, Balavoine G (2005) Caudal and even-skipped in the annelid Platynereis dumerilii and the ancestry of posterior growth. Evol Dev 7: 574–587. 10.1111/j.1525-142X.2005.05061.x 16336411

[68] Brulfert A, Monnot MJ, Geraudie J (1998) Expression of two even-skipped genes eve1 and evx2 during zebrafish fin morphogenesis and their regulation by retinoic acid. Int J Dev Biol 42: 1117–1124. 9879709

[69] Tiwari N, Tiwari Vijay K, Waldmeier L, Balwierz Piotr J, Arnold P, et al Sox4 Is a Master Regulator of Epithelial-Mesenchymal Transition by Controlling Ezh2 Expression and Epigenetic Reprogramming. Cancer Cell 23: 768–783. 10.1016/j.ccr.2013.04.020 23764001

[70] Myohara M, Niva CC, Lee JM (2006) Molecular approach to annelid regeneration: cDNA subtraction cloning reveals various novel genes that are upregulated during the large-scale regeneration of the oligochaete, Enchytraeus japonensis. Dev Dyn 235: 2051–2070. 10.1002/dvdy.20849 16724321

[71] Cho S-J, Lee MS, Tak ES, Lee E, Koh KS, et al (2009) Gene expression profile in the anterior regeneration of the earthworm using expressed sequence tags. Bioscience, biotechnology, and biochemistry 73: 29–34. 10.1271/bbb.80391 19129665

[72] Nyberg KG, Conte MA, Kostyun JL, Forde A, Bely AE (2012) Transcriptome characterization via 454 pyrosequencing of the annelid Pristina leidyi, an emerging model for studying the evolution of regeneration. BMC Genomics 13: 287 10.1186/1471-2164-13-287 22747785 PMC3464666

[73] Foronda M, Martinez P, Schoeftner S, Gomez-Lopez G, Schneider R, et al (2014) Sox4 links tumor suppression to accelerated aging in mice by modulating stem cell activation. Cell Rep 8: 487–500. 10.1016/j.celrep.2014.06.031 25043184 PMC4905521

[74] Vidricaire G, Jardine K, McBurney MW (1994) Expression of the Brachyury gene during mesoderm development in differentiating embryonal carcinoma cell cultures. Development 120: 115–122. 8119120 10.1242/dev.120.1.115

[75] McLean KE, Vickaryous MK (2011) A novel amniote model of epimorphic regeneration: the leopard gecko, Eublepharis macularius. BMC Dev Biol 11: 50 10.1186/1471-213X-11-50 21846350 PMC3180301

[76] Myers MW, Lazzarini RA, Lee VM, Schlaepfer WW, Nelson DL (1987) The human mid-size neurofilament subunit: a repeated protein sequence and the relationship of its gene to the intermediate filament gene family. EMBO J 6: 1617–1626. 3608989 10.1002/j.1460-2075.1987.tb02409.x PMC553533

[77] Yuan A, Rao MV, Nixon RA (2012) Neurofilaments at a glance. Journal of cell science 125: 3257–3263. 10.1242/jcs.104729 22956720 PMC3516374

[78] Wang H, Wu M, Zhan C, Ma E, Yang M, et al (2012) Neurofilament proteins in axonal regeneration and neurodegenerative diseases. Neural Regen Res 7: 620–626. 10.3969/j.issn.1673-5374.2012.08.010 25745454 PMC4346988

[79] Taniuchi M, Clark HB, Schweitzer JB, Johnson EM Jr. (1988) Expression of nerve growth factor receptors by Schwann cells of axotomized peripheral nerves: ultrastructural location, suppression by axonal contact, and binding properties. J Neurosci 8: 664–681. 2828568 10.1523/JNEUROSCI.08-02-00664.1988 PMC6569302

[80] Kassabov SR, Choi YB, Karl KA, Vishwasrao HD, Bailey CH, et al (2013) A single Aplysia neurotrophin mediates synaptic facilitation via differentially processed isoforms. Cell Rep 3: 1213–1227. 10.1016/j.celrep.2013.03.008 23562154 PMC4045214

[81] Varki A, Schauer R (2009) Sialic acids.

[82] Takahashi K, Mitoma J, Hosono M, Shiozaki K, Sato C, et al (2012) Sialidase NEU4 hydrolyzes polysialic acids of neural cell adhesion molecules and negatively regulates neurite formation by hippocampal neurons. J Biol Chem 287: 14816–14826. 10.1074/jbc.M111.324186 22393058 PMC3340223

[83] Varki A, Gagneux P (2012) Multifarious roles of sialic acids in immunity. Ann N Y Acad Sci 1253: 16–36. 10.1111/j.1749-6632.2012.06517.x 22524423 PMC3357316

[84] Surolia I, Pirnie SP, Chellappa V, Taylor KN, Cariappa A, et al (2010) Functionally defective germline variants of sialic acid acetylesterase in autoimmunity. Nature 466: 243–247. 10.1038/nature09115 20555325 PMC2900412

[85] Cariappa A, Takematsu H, Liu H, Diaz S, Haider K, et al (2009) B cell antigen receptor signal strength and peripheral B cell development are regulated by a 9-O-acetyl sialic acid esterase. J Exp Med 206: 125–138. 10.1084/jem.20081399 19103880 PMC2626685

[86] Miyagi T, Yamaguchi K (2012) Mammalian sialidases: physiological and pathological roles in cellular functions. Glycobiology 22: 880–896. 10.1093/glycob/cws057 22377912

[87] Dvorak J, Mancikova V, Pizl V, Elhottova D, Silerova M, et al (2013) Microbial environment affects innate immunity in two closely related earthworm species Eisenia andrei and Eisenia fetida. PLoS One 8: e79257 10.1371/journal.pone.0079257 24223917 PMC3815151

[88] Boros A, Reglodi D, Herbert Z, Kiszler G, Nemeth J, et al (2008) Changes in the expression of PACAP-like compounds during the embryonic development of the earthworm Eisenia fetida. J Mol Neurosci 36: 157–165. 10.1007/s12031-008-9102-6 18607777

[89] Varhalmi E, Somogyi I, Kiszler G, Nemeth J, Reglodi D, et al (2008) Expression of PACAP-like compounds during the caudal regeneration of the earthworm Eisenia fetida. J Mol Neurosci 36: 166–174. 10.1007/s12031-008-9125-z 18622585

[90] Huang J, Xu Q, Sun ZJ, Tang GL, Su ZY (2007) Identifying earthworms through DNA barcodes. Pedobiologia 51: 301–309.

[91] Harper GL, Cesarini S, Casey SP, Morgan AJ, Kille P, et al (2006) Microsatellite markers for the earthworm Lumbricus rubellus. Molecular Ecology Notes 6: 325–327.

[92] Velavan T, Schulenburg H, Michiels NK (2007) Development and characterization of novel microsatellite markers for the common earthworm (Lumbricus terrestris L.). Molecular Ecology Notes 7: 1060–1062.

[93] King RA, Tibble AL, Symondson WO (2008) Opening a can of worms: unprecedented sympatric cryptic diversity within British lumbricid earthworms. Mol Ecol 17: 4684–4698. 10.1111/j.1365-294X.2008.03931.x 18992008

[94] Heethoff M, Etzold K, Scheu S (2004) Mitochondrial COII sequences indicate that the parthenogenetic earthworm Octolasion tyrtaeum (Savigny 1826) constitutes of two lineages differing in body size and genotype. Pedobiologia 48: 9–13.

