# Supplementary material for: Large scale changes in the transcriptome of Eisenia fetida during regeneration
Source: PLoS One. 2018 Sep 27;13(9):e0204234. doi: 10.1371/journal.pone.0204234 (PMC6160089; doi:10.1371/journal.pone.0204234)
Supplement: S1 Table — (DOCX) [file pone.0204234.s001.docx]

S1 Table: Primers used in this study

| **Primer Name** | **Sequence** | |
| --- | --- | --- |
| Ef SIALIDASE1 FP\|RP | CGGAATTGCGGAAACTCACA | CATCGACGTCATTGCACAGTC |
| Ef SIALIDASE2 FP\|RP | AGTCCAACTGGGGAGGAA | GAGAAGGTCCATTTGGTGGA |
| Ef GAPDH FP\|RP | GACAGCACCCATGGACAGTTCAAG | TTCTATGACATACTCCGCACCCAC |
| Ef ProNGF FP\|RP | TGTCTACATATGTTACCATCGACCAGGCATAGG | TATAATCTCGAGACCTTTGACCTGTGACCGG |
| Ef SOX4 INSITU FP\|RP | CAGGGAGTACCCGGACTACA | CCACGAGTCACTTACCAGCA |
