# Supplementary material for: Large scale changes in the transcriptome of Eisenia fetida during regeneration
Source: PLoS One. 2018 Sep 27;13(9):e0204234. doi: 10.1371/journal.pone.0204234 (PMC6160089; doi:10.1371/journal.pone.0204234)
Supplement: S2 Fig — (DOCX) [file pone.0204234.s005.docx]

S2 Fig: Nerve Growth Factor from *E. fetida*

**SIGNAL PEPTIDE (predicted)**

acNGF MFPRTFPGLLISWAVILLSLQLLDAS

efNGF MIATASTILTTCLVLVQIVKG

hsNGF MSMLFYTLITAFLIGIQA

mmNGF MSMLFYTLITAFLIGVQA

**Pro-NGF region**

acNGF

DSVLSLTEGGSDPEADDVLGSDPGCSFPCVSADREDTDRDVLEPEDNYDTDTEPVDQESE

NEVPRPDQSITSDMTSHHKGPNMTPQDKGQSDTEGLYFILKSNRQPVTWKEPTNGRAAHT

SAKPGPVTSLGQD

efNGF

LPSTRHR**K**SASDGAWIPSQAAAMKQQLLLQLRQDNSLAMPISSDERAVISTSWPSEPPMS

LRFEASSGISEATLNATRMGDERAVISTSWPSEPPMSLRFEASSGISEATLNATRMG

hsNGF

EPHSESNVPAGHTIPQAHWTKLQHSLDTALRRA**R**SAPAAAIAARVAGQTRNITVDPRLFK

KRRLRSPRVLFSTQPPREAADTQDLDFEVGGAAPFN

mmNGF

EPYTDSNVPEGDSVPEAHWTKLQHSLDTALRRA**R**SAPTAPIAARVTGQTRNITVDPRLFK

KRRLHSPRVLFSTQPPPTSSDTLDLDFQAHGTIPFN

**Mature NGF**

Furin Cleavage site

#

acNGF KNEVRKK**R**SFD--------FVESEPVCPFQSRWVPLTHAR-DVHNRLVHVIQPGNLNDST

efNGF KRSPRST**R**SFHQPLAQAISSSSLVPVCESVSRWVKLTEVEDLWENRVTVVQQIDNGSGSL

hsNGF -RTHRSK**R**SSSH----PIFHRGEFSVCDSVSVWVGDKTTATDIKGKEVMVLGEVNINNSV

mmNGF -RTHRSK**R**SSTH----PVFHMGEFSVCDSVSVWVGDKTTATDIKGKEVTVLAEVNINNSV

||||||||.||*..**||||||||||||||||**|||*|**||.|.||||.|:|.|*||||*|.|*|

acNGF -AQWFRTVTCKEEDNQYEPSCPMCCRGIDSRRFHSMCRTTVTFVKAYGYEMTSTYQPVWP

efNGF VDQFFYETYCSRP-AGASADRPPSCIGTDTVNYDSVCLEKYVYVLGKVYYDE-------A

hsNGF FKQYFFETKCRDP-NP----VDSGCRGIDSKHWNSYCTTTHTFVK--ALTMD-------G

mmNGF FRQYFFETKCRAS-NP----VESGCRGIDSKHWNSYCTTTHTFVK--ALTTD-------E

|||||||||*:*||.|*||||||||||||||*|*|*:|.:.*|*||.|.:*||||||||||||||||

acNGF RIDDWHWIQVNASCTCNISPVHGRG--------

efNGF REERWTYVKIRASCNCAVIRKTSRRSRRSQVKG

hsNGF KQAAWRFIRIDTACVCVLSRKAVRRA-------

mmNGF KQAAWRFIRIDTACVCVLSRKATRRG-------

|||||||:|||*|::::|::*|*|:|||||*|||||||||
